# Association of HIF1α, BNIP3, and BNIP3L with Hypoxia-Related Metabolic Stress in Metabolic Syndrome

**DOI:** 10.3390/medicina62010166

**Published:** 2026-01-14

**Authors:** Tuğba Raika Kıran, Lezan Keskin, Mehmet Erdem, Zeynep Güçtekin, Feyza İnceoğlu

**Affiliations:** 1 Department of Medical Biochemistry, Faculty of Medicine, Malatya Turgut Özal University, Malatya 44210, Turkey; mehmet.erdem@ozal.edu.tr; 2 Department of Endocrinology, Faculty of Medicine, Malatya Turgut Özal University, Malatya 44210, Turkey; lezan.keskin@ozal.edu.tr; 3 Department of Education Unit, Malatya Turgut Özal University Education and Research Hospital, Malatya 44000, Turkey; zeynep.guctekin@saglik.gov.tr; 4 Department of Biostatistics, Faculty of Medicine, Malatya Turgut Özal University, Malatya 44210, Turkey; feyza.inceoglu@ozal.edu.tr

**Keywords:** metabolic syndrome, HIF1α, BNIP3, BNIP3L, hypoxia, mitophagy, mitophagy-related signaling, metabolic stress

## Abstract

*Background and Objectives*: Metabolic syndrome (MetS) is a complex condition marked by insulin resistance, central obesity, dyslipidemia, and chronic inflammation. Emerging evidence highlights the roles of hypoxia and mitochondrial stress in its pathophysiology. Hypoxia-inducible factor-1 alpha (HIF1α) and the mitophagy-associated proteins BNIP3 and BNIP3L are key components of hypoxia-responsive mitochondrial stress signaling. This study aimed to evaluate the circulating levels of HIF1α, BNIP3, and BNIP3L in MetS and to explore their associations with metabolic and inflammatory parameters. *Materials and Methods*: Serum concentrations of HIF1α, BNIP3, and BNIP3L were measured by ELISA in 40 patients with MetS and 40 age and sex-matched controls. Biochemical, hematological, and anthropometric parameters were assessed, and receiver operating characteristic (ROC) analyses were performed to evaluate diagnostic performance. *Results*: Serum levels of HIF1α, BNIP3, and BNIP3L levels were significantly higher in MetS patients compared with controls (*p* = 0.001). ROC analysis demonstrated strong diagnostic potential, particularly for BNIP3 (AUC = 0.928), followed by HIF1α (AUC = 0.885) and BNIP3L (AUC = 0.770). These markers showed significant associations with metabolic indicators such as BMI, fasting glucose, triglycerides, and inflammatory markers. *Conclusions*: The coordinated upregulation of circulating HIF1α, BNIP3, and BNIP3L in MetS is associated with metabolic dysregulation and systemic inflammation, reflecting alterations in hypoxia-responsive mitophagy-associated signaling rather than direct functional impairment of mitophagy. These findings support the potential relevance of these markers as indicators of metabolic stress in MetS. Further tissue-based and mechanistic studies are warranted to clarify their role in disease pathophysiology.

## 1. Introduction

Metabolic Syndrome (MetS) has emerged as a significant public health concern over the past three decades, with its prevalence rising globally due to multiple contributing factors. The prevalence of MetS is increasing globally, with various factors contributing to this rise. MetS is more prevalent in the Middle East and North America, with older men and younger women experiencing higher rates of diagnosis [1,2]. In a meta-analysis, the overall prevalence of MetS in Türkiye was reported as 32.9%, with rates of 38.3% in women and 26.8% in men [3]. MetS is characterized by the aggregation of three or more of the following five criteria: high blood pressure, dysglycemia, high plasma triglycerides, low high-density lipoprotein cholesterol, and increased waist circumference [4,5]. The presence of MetS significantly elevates the risk of developing serious health conditions, including type 2 diabetes, cardiovascular diseases, and stroke [6]. The underlying determinants of MetS are multifactorial, encompassing genetic predispositions, lifestyle factors such as sedentary behavior and poor dietary habits, and pre-existing conditions like gestational diabetes and polycystic ovary syndrome [7,8]. These factors collectively contribute to the development of insulin resistance, along with increased body mass and hypertension, which are pivotal in predisposing individuals to MetS and are closely linked to cardiometabolic risk [9,10].

Adipocyte hypertrophy is a hallmark of obesity and is associated with an increased risk of developing MetS, which encompasses a cluster of conditions including hypertension, dyslipidemia, hyperglycemia, and central obesity [11]. Enlarged adipocytes contribute to adipose tissue dysfunction, characterized by hypoxia due to insufficient blood supply, along with inflammation, reduced insulin sensitivity, impaired metabolic function, and increased adipokine secretion. Adipocytes that have increased in size often undergo hypoxic conditions because they do not have enough blood supply to support their larger size. This lack of oxygen can trigger hypoxia-inducible factors (HIFs), causing an increase in the production of proinflammatory cytokines and exacerbating insulin resistance [12,13]. Hypoxia-inducible factor 1 alpha (HIF1α), a subunit of the transcription factor HIF1, is a master regulator of the body’s adaptation to hypoxia. HIF1 plays a significant role in the physiological processes of adipocytes, such as differentiation, lipid metabolism, and the production of lactate. When adipocytes become enlarged, they undergo hypoxia, leading to the activation of HIF1α expression [14,15].

Under hypoxic conditions, activated HIF1α controls the transcription of many genes that are sensitive to hypoxia. Most of these genes are involved in regulating energy and oxygen levels, including glucose metabolism and oxidative phosphorylation [16]. In addition, hypoxia is an important factor that triggers mitophagy and HIF1α plays important roles in the regulation of this pathway [17]. Mitophagy is a selective autophagy process that eliminates damaged or dysfunctional mitochondria to preserve cellular homeostasis and prevent reactive oxygen species (ROS) accumulation, and it is regulated by key proteins such as Parkin, PTEN-induced putative kinase 1 (PINK1), FUN14 domain containing 1 (FUNDC1), Bcl-2/adenovirus E1B 19 kDa interacting protein 3 (BNIP3), and BNIP3-like (BNIP3L or NIX) [18,19]. These proteins are key regulators of mitophagy, the selective degradation of damaged mitochondria to maintain cellular homeostasis. BNIP3 and BNIP3L are members of the BH3-only subgroup of the Bcl-2 family of proteins, which are known to play critical roles in regulating apoptosis and autophagy. The expression of BNIP3 is primarily regulated at the transcriptional level by HIFs, particularly HIF1α. Under hypoxic conditions, HIF1α binds to hypoxia response elements (HRE) in the BNIP3/BNIP3L promoter, leading to their transcriptional activation [20,21,22]. BNIP3 and BNIP3L regulate mitophagy under hypoxic conditions through phosphorylation and dimerization, while also being modulated transcriptionally via HIF1α [23]. Additionally, BNIP3 has been shown to promote PINK1-Parkin-mediated mitophagy [23], while the downregulation of Sirtuin 3 (Sirt3) suppresses BNIP3/BNIP3L pathway-driven mitophagy [24]. Activation of BNIP3L induces mitochondrial depolarization, triggers mitophagy, and contributes to impaired insulin signaling [25]. These findings highlight the importance of BNIP3 and BNIP3L pathways in regulating mitophagy in MetS; however, the precise mechanisms require further exploration [17].

Despite extensive experimental evidence linking hypoxia to BNIP3/BNIP3L-dependent mitophagy, clinical data simultaneously evaluating hypoxia-responsive mitophagy-related markers in metabolic syndrome remain limited. In particular, the systemic reflection of hypoxia-driven mitophagy signaling, as assessed by circulating levels of HIF1α together with its downstream receptors BNIP3 and BNIP3L, has not been comprehensively examined in patients with metabolic syndrome. Therefore, this study is original in jointly profiling these hypoxia-related mitophagy-associated markers in a clinical MetS cohort and relating them to metabolic and inflammatory parameters. Accordingly, the main research question addressed by this study was whether hypoxia-related mitophagy signaling, reflected by circulating levels of HIF1α and its downstream mitophagy receptors BNIP3 and BNIP3L, is altered in patients with metabolic syndrome and whether these alterations are associated with metabolic and inflammatory disturbances, thereby supporting their potential relevance as systemic biomarkers of metabolic stress.

## 2. Materials and Methods

### 2.1. Study Design and Participants

This study was conducted between October 2024 and January 2025 at the Department of Endocrinology Faculty of Medicine Malatya Turgut Özal University, Malatya, Turkey. The patient and control groups in the study were composed of volunteers who applied to Faculty of Medicine Malatya Turgut Özal University. Forty individuals aged 18–65 years diagnosed with MetS according to NCEP ATP III were included in the study [26]. Individuals with Type 1 diabetes, cancer, chronic gastrointestinal diseases, pregnant or breastfeeding women, those who had undergone antibiotic treatment within one month prior to the study, individuals experiencing weight loss, those following a dietary treatment, those using blood glucose or lipid-regulating medications other than metformin, and individuals taking nutritional supplements that could affect metabolic parameters were not included in the study. Forty individuals of the same ethnic origin and with similar demographic characteristics, aged between 18 and 65 years, who met the exclusion criteria and had no diseases, were included in the control group.

### 2.2. Data Collection

Detailed demographic and lifestyle information such as age, body weight (BW), body height (BH), waist circumference (WC), hip circumference (HC), systolic and diastolic blood pressure (SBP and DBP), gender, smoking status, alcohol consumption, disease history, and diet history were obtained from the individuals included in the study. To measure SBP and DBP, volunteers rested for at least 10 min under appropriate conditions. Then, SBP and DBP were measured twice on the left arm using a digital sphygmomanometer (Omron M3, Omron Corporation, Tokyo, Japan) while the volunteers were in a seated position. BW was measured with a body composition analyzer (BC-420MA, Tanita, Tokyo, Japan). BH was measured using a stadiometer. The body mass index (BMI) was determined by dividing BW (kg) by the square of BH (m^2^). WC was measured with a standard tape at the midpoint between the lowest rib and the iliac crest. For the measurements, individuals removed their shoes, and the measurements were taken while wearing as little clothing as possible. HC was measured using a non-elastic measuring tape while the participant was in a standing position. The measurement was taken at the widest part of the hips, ensuring that the tape remained parallel to the floor and without excessive tension. All measurements were performed by a trained evaluator to maintain consistency and minimize interobserver variability.

### 2.3. Sample Collection and Biochemical Analysis

Early in the morning, after an overnight fast, blood samples were collected by a single specialist phlebotomist, from the brachial veins of all volunteers into two gel separator tubes (serum collection) and one K_2_EDTA tube. After the collection process, all tubes were properly transported to the biochemistry laboratory for routine analysis. Hemogram analyses (HG, erythrocytes, leukocytes, lymphocytes, monocytes, neutrophils, platelets, and MCV) and HbA1c measurements in K_2_EDTA tubes were performed using a hemogram analyzer (Sysmex Corporation, XN-10, Kobe, Japan) and an automatic glycohemoglobin analyzer (Arkray, Adams A1c HA-8180V, Kyoto, Japan), respectively. The serum collection tubes were left for 20–30 min to allow clotting. Once coagulation was complete, the serum collection tubes were centrifuged at 1800 g for 10 min. First serum collection tube was used for biochemical analysis (FG, TG, TC, HDL LDL, urea, creatinine, uric acid, albumin, ALT, AST, CRP) and hormone analysis (FI, TSH, T3, and T4). The analyses were conducted using a biochemistry analyzer (Abbott Architect c16000, Chicago, IL, USA) and a hormone analyzer (Roche Diagnostics Cobas E601, Tokyo, Japan), respectively. The HOMA-IR index was calculated to evaluate insulin resistance. The HOMA-IR value was determined using the following formula: Fasting plasma insulin (μU/mL) multiplied by fasting plasma glucose (mg/dL), and the result was then divided by 405. Serum samples in the second serum collection tube (obtained at the end of centrifugation) were transferred to 1.5 mL micro-volume tubes. These serum samples were placed in a −80 °C deep freezer for HIF1α, BNIP3, and BNIP3L analyses and stored there until the analyses were conducted.

Serum HIF-1α (Bioassay Technology Laboratory, Cat. No: E0422Hu, Shanghai, China), BNIP3 (ELK Biotechnology, Catalog No: ELK8447, Sugar Land, TX, USA), and BNIP3L (ELK Biotechnology, Catalog No: ELK9461, Sugar Land, TX, USA) levels were measured using commercially available Enzyme-Linked Immunosorbent Assay (ELISA) kits in accordance with the manufacturers’ protocols. All measurements were performed in duplicate using the same reagent lot to minimize inter-assay variability, and sample analysis was conducted in a blinded manner. The intra-assay coefficient of variation was maintained below 10%, as specified by the manufacturers. According to the manufacturers’ datasheets, the analytical measurement ranges of the ELISA kits were 0.05–15 ng/mL for HIF-1α and 0.16–10 ng/mL for both BNIP3 and BNIP3L, allowing reliable quantification of these proteins within the expected concentration ranges in human serum. Additionally, the reported analytical sensitivities were 0.01 ng/mL for HIF-1α, 0.095 ng/mL for BNIP3, and 0.094 ng/mL for BNIP3L.

### 2.4. Statistical Analysis

The data analyses for this study were conducted using the SPSS (Statistical Program for Social Sciences) version 25. The conformity of the data to a normal distribution was assessed using the Kolmogorov–Smirnov test. Descriptive statistics, including mean, standard deviation, frequency, and percentage values, were used to summarize the data. A significance level (*p*) of 0.05 was considered for all comparative tests. As the variables were found to follow a normal distribution (*p* > 0.05), parametric test methods were employed for further analyses. Comparisons between two independent groups were performed using the *t*-test, given that the normality assumption was satisfied. To determine the cut-off point for a specific measurement value, ROC analysis was conducted, and the indices were calculated. The relationships between numerical variables were examined using the Pearson correlation coefficient. To control for the potential confounding effect of body mass index (BMI) on group differences, BMI was included as a covariate and analysis of covariance (ANCOVA) was applied.

## 3. Results

### 3.1. Basic Demographic and Anthropometric Data

The mean ages of MetS patients and control subjects were 39.83 ± 13.22 and 38.77 ± 10.14 years, respectively, with no significant difference between the groups (*p* = 0.691). The mean body mass index (BMI) of MetS patients and controls was 33.77 ± 3.96 and 23.51 ± 2.72, respectively, with significantly higher values observed in the MetS group (*p* = 0.001). The mean waist circumference (WC) was 108.1 ± 8.89 in the MetS group and 77.53 ± 13.23 in the control group, also significantly higher in MetS (*p* = 0.001). Similarly, the mean hip circumference (HC) value of MetS patients and control subjects was 111.67 ± 11.17 and 90.65 ± 10.45, respectively (*p* = 0.001). The mean systolic blood pressure (SBP) was 128.25 ± 10.83 in MetS patients and 114.75 ± 7.16 in controls, with significantly higher values in the MetS group (*p* = 0.001). The mean diastolic blood pressure (DBP) was 82.5 ± 6.3 in MetS patients and 76.9 ± 12.85 in controls, also significantly higher in the MetS group (*p* = 0.016). Among MetS patients, 60% (*n* = 24) were female and 40% (*n* = 16) were male, while in the control group, 52.50% (*n* = 21) were female and 47.50% (*n* = 19) were male. No significant difference was observed between the groups in terms of gender distribution (*p* = 0.652). Among MetS patients, 60% (*n* = 24) were non-smokers, 30% (*n* = 12) were smokers, and 10% (*n* = 4) were former smokers, while in the control group, 65% (*n* = 26) were non-smokers, 35% (*n* = 14) were smokers. No significant difference was observed between the MetS and control groups in terms of smoking status (*p* = 0.120). Among MetS patients, 90% (*n* = 36) reported no alcohol consumption, while 10% (*n* = 4) consumed alcohol. In the control group, 97.50% (*n* = 39) reported no alcohol consumption, whereas 2.50% (*n* = 1) consumed alcohol. No significant difference was observed between the MetS and control groups in terms of alcohol consumption (*p* = 0.356) (Table 1).

### 3.2. Evaluation of Clinical Laboratory Parameters

Within the study, no statistically significant differences were observed between the MetS and control groups in terms of aspartate aminotransferase (AST), urea, creatinine, uric acid, albumin, hemoglobin (HG), erythrocyte, lymphocyte, monocyte, platelet, thyroid-stimulating hormone (TSH), free triiodothyronine (T3), and free thyroxine (T4) levels (*p* > 0.05) (Table 2). On the other hand, glycated hemoglobin (HbA1c), fasting glucose (FG), fasting insulin (FI), homeostatic model assessment of insulin resistance (HOMA-IR), high-density lipoprotein (HDL), total cholesterol (TC), triglyceride (TG), low-density lipoprotein (LDL), C-reactive protein (CRP), alanine aminotransferase (ALT), leukocyte, neutrophil, and mean corpuscular volume (MCV) levels were found to be statistically significantly different in the MetS group compared to the control group (*p* < 0.05) (Table 2).

### 3.3. Evaluation of HIF1α, BNIP3, and BNIP3L Levels

**Figure 1:**
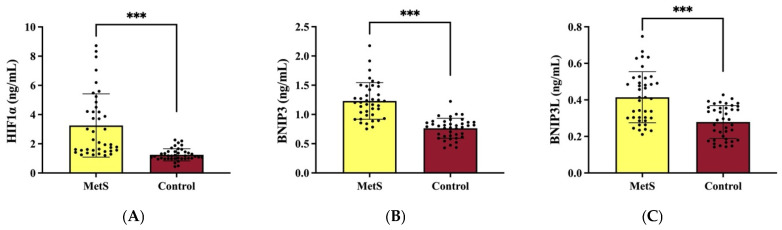
HIF1α (**A**), BNIP3 (**B**), and BNIP3L (**C**) analysis. Data expressed as mean ± S.D. *** *p* = 0.001 in comparison to control. HIF1α, hypoxia-inducible factor 1 alpha; BNIP3, Bcl-2/adenovirus E1B 19 kDa interacting protein 3; BNIP3L, BNIP3-like.

The mean HIF1α levels in MetS patients and controls were 3.25 ± 2.17 ng/mL and 1.25 ± 0.42 ng/mL, respectively, with significantly higher levels observed in the MetS group (*p* = 0.001) (Figure 1A). Similarly, BNIP3 levels were 1.23 ± 0.31 ng/mL in MetS patients and 0.76 ± 0.17 ng/mL in controls, again significantly elevated in the MetS group (*p* = 0.001) (Figure 1B). BNIP3L levels were 0.41 ± 0.14 ng/mL in MetS patients and 0.28 ± 0.09 ng/mL in controls, also significantly higher in the MetS group (*p* = 0.001) (Figure 1C).

### 3.4. Receiver Operating Characteristic (ROC) Curve Analysis

**Figure 2:**
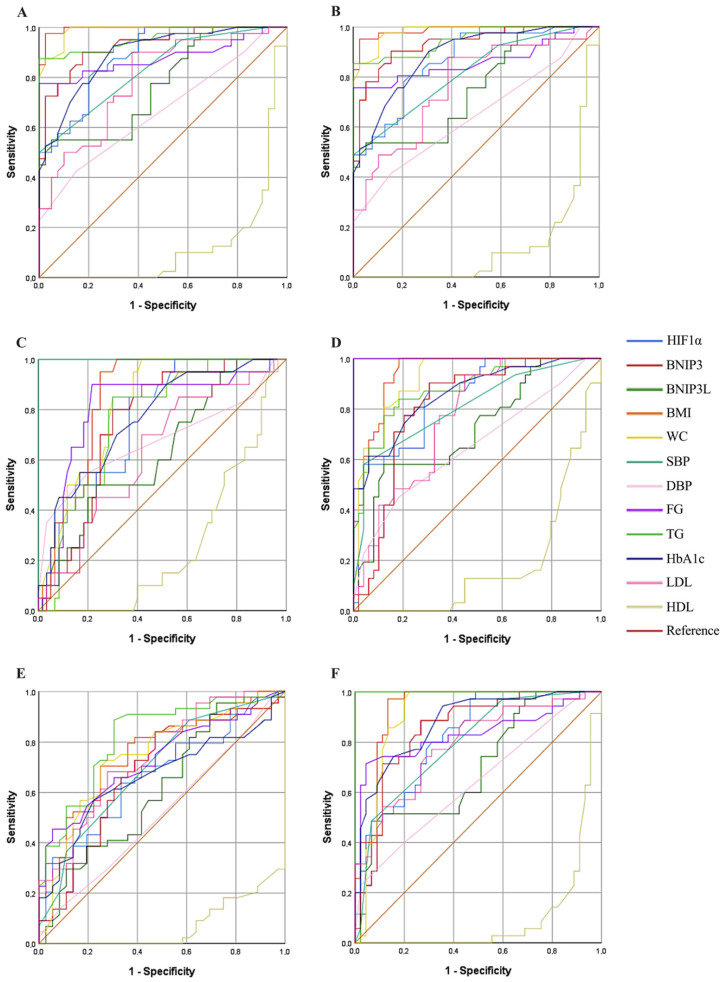
ROC analyses. Cut-off values for patient and control (**A**), presence or absence of abdominal obesity (**B**), low-high blood pressure (**C**), low-high fasting glucose (**D**), low-high HDL (**E**), and low-high triglycerides (**F**). Abdominal obesity: WC ≥ 102 cm in men and ≥88 cm in women; high TG ≥ 150 mg/dL; high fasting FG ≥ 100 mg/dL; low HDL: HDL < 40 mg/dL in men and <50 mg/dL in women; high blood pressure: SBP ≥ 130, DBP ≥ 85. HIF1α, hypoxia-inducible factor 1 alpha; BNIP3, Bcl-2/adenovirus E1B 19 kDa interacting protein 3; BNIP3L, BNIP3-like; BMI, body mass index; WC, waist circumference; SBP, systolic blood pressure; DBP, diastolic blood pressure; FG, fasting glucose; TG, triglyceride; HbA1c, glycated hemoglobin; LDL, low-density lipoprotein; HDL, high-density lipoprotein.

ROC analysis was conducted to determine the cut-off values of HIF1α, BNIP3, BNIP3L, BMI, WC, SBP, DBP, FG, TG, HbA1c, LDL, and HDL across six models (patient–control differentiation, abdominal obesity, blood pressure, FG, HDL, and TG). All AUC values reported were statistically significant unless otherwise specified (*p* < 0.05) (Figure 2). Six models were prepared and calculated: The first model compared the patient and control groups, the second model pertained to abdominal obesity, the third model to blood pressure, the fourth model to FG, the fifth model to HDL, and the sixth model to TG. HIF1α demonstrated strong discriminative ability, with an AUC of 0.885 (95% CI: 0.816–0.954) for patient-control differentiation, 0.866 (95% CI: 0.789–0.942) for abdominal obesity, 0.848 (95% CI: 0.764–0.932) for FG, 0.758 (95% CI: 0.651–0.865) for blood pressure, 0.664 (95% CI: 0.546–0.783) for HDL, and 0.825 (95% CI: 0.737–0.913) for TG. BNIP3 showed the highest predictive accuracy among serum biomarkers, with an AUC of 0.928 (95% CI: 0.873–0.982) for patient-control differentiation, 0.933 (95% CI: 0.880–0.986) for abdominal obesity, 0.747 (95% CI: 0.638–0.857) for blood pressure, 0.811 (95% CI: 0.714–0.908) for FG, 0.681 (95% CI: 0.560–0.801) for HDL, and 0.850 (95% CI: 0.765–0.936) for TG. BNIP3L exhibited moderate discriminative capacity, with AUC values of 0.770 (95% CI: 0.668–0.872) for patient-control, 0.754 (95% CI: 0.649–0.859) for abdominal obesity, 0.631 (95% CI: 0.494–0.768) for blood pressure, 0.720 (95% CI: 0.604–0.836) for FG, 0.612 (95% CI: 0.487–0.738) for HDL, and 0.699 (95% CI: 0.583–0.815) for TG. Anthropometric markers BMI and WC displayed exceptionally high predictive performance across all models. For BMI, the AUC was 0.994 (95% CI: 0.985–1.000) for patient-control, 0.987 (95% CI: 0.969–1.000) for abdominal obesity, 0.845 (95% CI: 0.763–0.928) for blood pressure, 0.944 (95% CI: 0.898–0.991) for FG, 0.755 (95% CI: 0.649–0.861) for HDL, and 0.933 (95% CI: 0.877–0.990) for TG. WC yielded similarly high AUC values: 0.987 (95% CI: 0.970–1.000), 0.982 (95% CI: 0.961–1.000), 0.818 (95% CI: 0.727–0.909), 0.922 (95% CI: 0.866–0.978), 0.744 (95% CI: 0.634–0.854), and 0.908 (95% CI: 0.837–0.980), respectively. SBP showed moderate to strong discriminative capacity, with AUCs of 0.833 (95% CI: 0.747–0.919) for patient-control, 0.815 (95% CI: 0.724–0.906) for abdominal obesity, 1.000 (95% CI: 1.000–1.000) for blood pressure, 0.810 (95% CI: 0.710–0.911) for FG, 0.698 (95% CI: 0.582–0.813) for HDL, and 0.797 (95% CI: 0.700–0.893) for TG. In contrast, DBP demonstrated consistently lower predictive performance across models, with AUCs of 0.666 (95% CI: 0.547–0.785), 0.645 (95% CI: 0.525–0.766), 0.675 (95% CI: 0.517–0.834), 0.654 (95% CI: 0.528–0.781), 0.518 (95% CI: 0.391–0.646), and 0.641 (95% CI: 0.517–0.764), respectively. Among metabolic parameters, FG (AUC range: 0.730–1.000), TG (0.763–1.000), and HbA1c (0.766–0.894) showed robust predictive performance, while LDL exhibited moderate values (0.632–0.791). HDL consistently had the lowest predictive capacity across models (AUC range: 0.071–0.260), reflecting its poor discriminatory power (Figure 2).

### 3.5. BMI-Adjusted Group Comparisons (ANCOVA Analysis)

To evaluate the potential confounding effect of BMI, analysis of covariance (ANCOVA) was performed with BMI included as a covariate. After adjustment for BMI, the group × BMI interaction was not statistically significant. Specifically, the interaction between group and BMI (Group * BMI) was not significant for any of the investigated parameters, including HIF-1α, BNIP3, and BNIP3L (*p* > 0.05). These findings indicate that the differences observed between the MetS and control groups were independent of BMI and remained evident after BMI adjustment (Table 3).

## 4. Discussion

This study investigated the relationship between hypoxia-related signaling involving HIF1α and the mitophagy-associated proteins BNIP3 and BNIP3L in patients with MetS. Given that mitophagy plays a critical role in mitochondrial quality control, alterations in hypoxia-related mitophagy signaling may be associated with metabolic imbalances observed in MetS. To date, no studies have comprehensively examined the combined behavior of circulating HIF1α, BNIP3, and BNIP3L in MetS, particularly in relation to metabolic and inflammatory parameters. Although these factors are individually implicated in hypoxia responses, mitochondrial homeostasis, and oxidative stress, their coordinated systemic profile in the context of MetS has remained largely unexplored. Collectively, our findings provide novel clinical insight into hypoxia-responsive mitophagy-related signaling in MetS without implying direct functional impairment of mitophagy.

Our study provides further evidence supporting the role of HIF1α in the pathophysiology of MetS by demonstrating its significantly elevated expression in individuals with MetS compared to healthy controls. Studies have demonstrated a significant upregulation of HIF1α expression in the adipose tissue of obese individuals compared to their lean counterparts, highlighting its role in the pathophysiology of MetS [27]. In agreement with previous findings [27,28], our results indicate that the overexpression of HIF1α is associated with metabolic dysregulation, including altered glucose metabolism and lipid homeostasis. The increased HIF1α expression observed in MetS patients may be a compensatory response to adipose tissue hypoxia, a common feature of obesity, which leads to mitochondrial dysfunction and impaired metabolic flexibility. Furthermore, our study reveals that hepatic HIF1α expression is significantly elevated in MetS patients, suggesting a possible association of liver-specific hypoxia-driven pathways to systemic metabolic disturbances. This aligns with previous reports indicating that while hepatic HIF1α deletion confers protection against obesity-induced glucose intolerance. In contrast, its sustained activation may exacerbate metabolic dysfunction [29]. The interplay between hepatic HIF1α, glucose metabolism, and insulin sensitivity warrants further investigation, as our findings reinforce the notion that liver-specific HIF1α modulation could be a promising therapeutic avenue for MetS management. Additionally, our study supports the hypothesis that excessive HIF1α activation contributes to adipose tissue remodeling. This includes the upregulation of glucose transporter 1 (GLUT1) and pyruvate dehydrogenase kinase 1 (PDK1), which are crucial for glucose metabolism and lipid storage, thereby influencing overall energy homeostasis, consistent with prior experimental findings [30,31]. The observed correlation between HIF1α overexpression and systemic inflammatory markers in MetS patients further suggests that HIF1α-driven inflammatory pathways may play a crucial role in exacerbating insulin resistance and lipid dysregulation. Interestingly, preclinical studies have also demonstrated that stabilization of HIF1α through prolyl hydroxylase domain (PHD) inhibition can result in paradoxical metabolic benefits, including reductions in BMI, lipid levels, and adipose tissue fibrosis [31]. This indicates that the metabolic consequences of HIF1α modulation are highly context-dependent. Therefore, therapeutic approaches should carefully differentiate between adaptive and maladaptive hypoxia responses in MetS.

Mitophagy is a selective mitochondrial quality control process responsible for the removal of damaged or dysfunctional mitochondria through autophagy. Increasing evidence indicates that hypoxia and oxidative stress are major upstream triggers of mitophagic signaling. Excessive ROS generation under metabolic stress stabilizes HIF-1α, which transcriptionally induces mitophagy receptors such as BNIP3 and BNIP3L. These receptors directly interact with LC3, thereby promoting mitochondrial targeting to the autophagic machinery. Recent experimental data have demonstrated that ROS-mediated HIF-1α activation enhances BNIP3L-LC3 binding and mitophagy, ultimately contributing to tissue injury under sustained stress conditions, such as toxin-induced myocardial damage [32]. Importantly, emerging regulatory mechanisms indicate that BNIP3/BNIP3L-dependent mitophagy requires tight control, as excessive or insufficient activation may disrupt mitochondrial homeostasis. In this regard, PPTC7 has been identified as a critical co-factor restricting BNIP3/BNIP3L-mediated mitophagy through ubiquitin-dependent regulatory pathways, highlighting that dysregulated mitophagy can be detrimental rather than protective [33]. Within this framework, the elevated circulating levels of HIF-1α, BNIP3, and BNIP3L observed in the present study likely reflect systemic activation of hypoxia-responsive mitophagy signaling in response to chronic metabolic stress. Although direct assessment of mitophagic flux was not performed, the coordinated upregulation of these markers supports the concept that maladaptive or dysregulated hypoxia-related mitophagy signaling may coexist with mitochondrial dysfunction and metabolic inflammation under conditions of chronic metabolic stress. The absence of a significant group * BMI interaction for HIF-1α, BNIP3, and BNIP3L indicates that the observed alterations are not merely a consequence of increased adiposity but rather reflect BMI-independent processes intrinsically associated with metabolic syndrome; a similar pattern was consistently observed across the other investigated variables.

The ROC analysis confirmed the strong discriminatory performance of HIF1α for distinguishing MetS patients from controls, with an AUC of 0.885, indicating good diagnostic accuracy. Similarly, a high AUC value (0.866) was observed in the abdominal obesity model, supporting an association between HIF1α expression and obesity-related metabolic stress. Subgroup ROC analyses for blood pressure and metabolic parameters showed moderate discriminatory performance (SBP AUC: 0.758; FG AUC: 0.848), and these findings should be interpreted as exploratory given the limited sample size. In addition, HIF1α exhibited a stronger association with triglyceride levels (AUC: 0.825) compared with HDL (AUC: 0.664), suggesting a differential relationship with lipid-related parameters rather than robust discriminatory ability for HDL. Collectively, these results indicate that HIF1α is associated with multiple metabolic features of MetS and may serve as a potential biomarker reflecting hypoxia-related metabolic stress. However, the ROC findings for secondary models should be interpreted cautiously and warrant validation in larger, independent cohorts.

Our study further supports the role of BNIP3 in the metabolic dysregulation observed in MetS by demonstrating a significant increase in BNIP3 expression in individuals with MetS compared to healthy controls. This finding aligns with previous reports indicating that BNIP3-mediated mitophagy plays a crucial role in oxidative stress modulation and mitochondrial quality control, both of which are critical in the progression of metabolic disorders [34,35]. The elevated levels of BNIP3 observed in MetS patients in our study may reflect a compensatory response to increased mitochondrial dysfunction and oxidative stress, a hallmark of insulin resistance and obesity-related metabolic disturbances. Moreover, our findings suggest that BNIP3 overexpression in MetS is associated with alterations in adipocyte function and remodeling, consistent with prior studies demonstrating the upregulation of BNIP3 during adipocyte differentiation [36,37]. This supports the notion that BNIP3 is involved in the adaptation of adipose tissue to metabolic stress by regulating mitochondrial turnover and energy homeostasis. However, excessive BNIP3 activation, particularly under prolonged metabolic stress, may contribute to adipocyte dysfunction, exacerbating insulin resistance and lipid dysregulation. The ROC analysis demonstrated a strong discriminative performance of BNIP3 in distinguishing MetS patients from healthy controls (AUC: 0.928). Similarly, a high AUC value was observed in the abdominal obesity model (AUC: 0.933), supporting an association between BNIP3 levels and obesity-related metabolic stress. Subgroup ROC analyses showed moderate discriminatory performance for blood pressure (AUC: 0.747) and fasting glucose (AUC: 0.811), and these findings should be interpreted as exploratory due to the limited sample size. In addition, BNIP3 exhibited a stronger association with triglyceride levels (AUC: 0.85) compared with HDL (AUC: 0.681), indicating differential relationships with lipid-related parameters rather than robust discrimination for HDL. Collectively, these results suggest that circulating BNIP3 is associated with multiple metabolic features of MetS and may serve as a potential biomarker reflecting systemic metabolic stress. However, the ROC findings for secondary models require cautious interpretation and validation in larger cohorts. Given its strong predictive value in multiple metabolic parameters, further investigation is warranted to explore its potential as a therapeutic target for metabolic disorders.

The BNIP3L has been shown to play a pivotal role in mitochondrial quality control, and its downregulation has been associated with increased susceptibility to metabolic disturbances [38,39]. Furthermore, studies indicate that the activation of BNIP3L can enhance mitochondrial fission and mitophagy, thereby improving glucose uptake in muscle cells, which is often impaired in MetS [25]. Our study demonstrates a significant upregulation of BNIP3L expression in MetS patients compared to healthy controls, reinforcing its role in mitophagy regulation and metabolic dysfunction. BNIP3L, a key mitochondrial stress sensor, has been previously implicated in hypoxia-induced autophagy, mitochondrial clearance, and cellular adaptation to metabolic stress. The observed increase in BNIP3L levels in MetS patients suggests an adaptive response to mitochondrial dysfunction, which is a hallmark of insulin resistance and obesity-related metabolic dysregulation. The ROC analysis indicated a moderate discriminatory performance of BNIP3L in distinguishing MetS patients from controls (AUC: 0.77). Similar moderate performance was observed in the abdominal obesity model (AUC: 0.754), supporting an association between BNIP3L levels and obesity-related metabolic stress. Subgroup ROC analyses showed weaker discriminatory performance for blood pressure (AUC: 0.631) and FG (AUC: 0.72), and these findings should be interpreted cautiously given the limited sample size. In addition, BNIP3L exhibited mild associations with triglyceride (AUC: 0.699) and HDL levels (AUC: 0.612), indicating limited discriminatory ability for lipid-related parameters. Collectively, these results suggest that circulating BNIP3L is modestly associated with metabolic features of MetS and may reflect systemic metabolic stress rather than robust predictive capacity. Further validation in larger cohorts is warranted. Additionally, our study highlights the potential link between BNIP3- and BNIP3L-associated hypoxia-responsive mitochondrial stress pathways and systemic metabolic inflammation in MetS, further reinforcing the role of HIF1α in this process. BNIP3 and BNIP3L have been shown to regulate mitochondrial turnover in response to oxidative stress, and their excessive activation may lead to pro-inflammatory signaling, thereby exacerbating chronic low-grade inflammation, a hallmark of MetS. Our findings indicate significantly elevated systemic inflammatory markers, including CRP, and leukocyte count in MetS patients, consistent with an inflammatory metabolic state.

**Figure 3:**
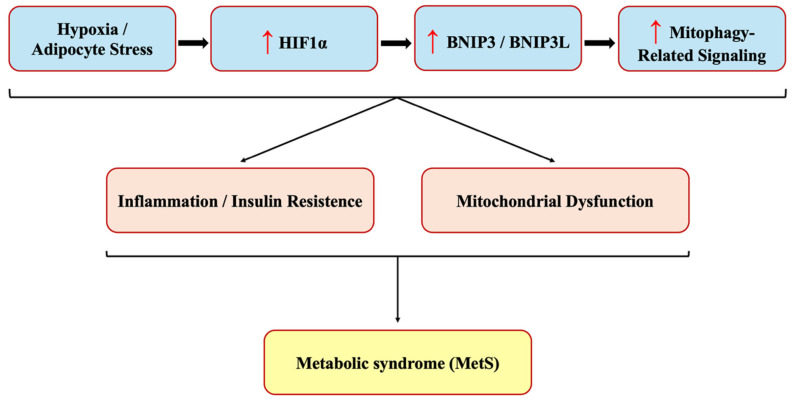
Hypothesized model illustrating the potential involvement of HIF1α-related signaling in MetS. Under hypoxic or adipocyte stress conditions, increased HIF1α expression may be associated with elevated levels of the mitophagy-related proteins BNIP3 and BNIP3L. Although BNIP3 and BNIP3L are known regulators of mitochondrial quality control, the present study does not directly assess mitophagic activity or mitochondrial function. Therefore, the proposed links to mitochondrial stress, inflammation, and insulin resistance are inferred from observed serum associations and existing literature and should be interpreted as a conceptual framework rather than a validated mechanism. MetS, metabolic syndrome; HIF1α, hypoxia-inducible factor 1 alpha; BNIP3, Bcl-2/adenovirus E1B 19 kDa interacting protein 3; BNIP3L, BNIP3-like. Red arrows indicate increased circulating (serum) levels of the corresponding proteins.

Our study demonstrated a significant upregulation of BNIP3, BNIP3L, and HIF1α expression in MetS patients, supporting the hypothesis that hypoxia-driven alterations in mitophagy-related signaling are associated with systemic metabolic dysfunction in MetS. HIF1α, as a central regulator of cellular adaptation to hypoxia, plays a key role in metabolic reprogramming and inflammation. Its increased expression in MetS suggests an adaptive but potentially maladaptive response to metabolic stress, promoting insulin resistance, lipid dysregulation, and endothelial dysfunction. The concurrent upregulation of BNIP3 and BNIP3L, both transcriptional targets of HIF1α, further suggests that HIF1α-driven mitophagy alterations may be involved in obesity-related metabolic disturbances (Figure 3). Collectively, these findings underscore the interconnected roles of HIF1α, BNIP3, and BNIP3L in driving systemic metabolic inflammation in MetS, emphasizing their potential as biomarkers and therapeutic targets for metabolic disorders. It should be noted that our study design is observational; therefore, causality between elevated HIF1α, BNIP3, and BNIP3L levels and metabolic dysfunction cannot be definitively established. While our findings strongly support an association, it remains unclear whether these proteins act as causal drivers or are consequences of metabolic stress. Future studies should incorporate prospective cohort designs to evaluate their predictive role in the development of MetS. Moreover, mechanistic investigations using adipose and hepatic tissue biopsies, as well as cellular models exposed to hypoxia, will be valuable to determine whether the observed serum elevations correspond to functional alterations at the tissue level. Further investigations are warranted to elucidate the mechanistic interactions between hypoxia, mitophagy, and inflammation, particularly in the context of MetS progression and targeted interventions.

The limited sample size and single-center design may restrict the generalizability of the findings. Furthermore, the sample was drawn from a specific demographic group, which may limit the applicability of the results to other ethnic groups or age populations. Potential confounding factors -particularly diet, physical activity levels, and genetic predisposition- were not accounted for. Additionally, only serum levels were assessed; the study did not include tissue-level analyses such as gene or protein expression profiling in metabolically active organs (e.g., adipose tissue or liver), which could have provided more precise mechanistic insights. Moreover, given the observational, case–control design of the study, causal relationships between elevated HIF1α, BNIP3, and BNIP3L levels and metabolic dysfunction cannot be established. While the associations observed are statistically significant and biologically plausible, it remains unclear whether these proteins act as causal drivers or merely represent consequences of metabolic disturbances.

## 5. Conclusions

This study provides clinically relevant insight into the association of HIF1α and the mitophagy-associated proteins BNIP3 and BNIP3L with hypoxia-related metabolic dysregulation and systemic inflammation in metabolic syndrome. The coordinated upregulation of these circulating markers suggests the presence of altered hypoxia-responsive mitophagy-related signaling at the systemic level, rather than direct impairment of mitophagic function. The observed relationships with metabolic and inflammatory parameters, together with ROC-based analyses, support the potential utility of HIF1α, BNIP3, and BNIP3L as biomarkers reflecting metabolic stress in MetS.

Given the observational nature of the study, causal inferences cannot be made. Nevertheless, these findings extend existing experimental evidence into a clinical setting and provide a translational framework for future tissue-based and mechanistic studies aimed at clarifying the role of hypoxia-driven mitochondrial stress pathways in metabolic syndrome.

## Figures and Tables

**Table 1:** Comparison of basic demographic and anthropometric variables by groups.

Variable	Group		*p* Value
	MetS (Mean ± SD)	Control (Mean ± SD)	
Age	39.83 ± 13.22	38.77 ± 10.14	0.691
BMI (kg/m^2^)	33.77 ± 3.96	23.51 ± 2.72	**0.001**
WC (cm)	108.1 ± 8.89	77.53 ± 13.23	**0.001**
HC (cm)	111.67 ± 11.17	90.65 ± 10.45	**0.001**
SBP (mmHg)	128.25 ± 10.83	114.75 ± 7.16	**0.001**
DBP (mmHg)	82.5 ± 6.3	76.9 ± 12.85	**0.016**
Gender (*n*%)			
Female	24 (60.00%)	21 (52.50%)	0.652
Male	16 (40.00%)	19 (47.50%)	
Smoking status (*n*%)			
No	24 (60.00%)	26 (65.00%)	0.120
Yes	12 (30.00%)	14 (35.00%)	
Quit	4 (10.00%)	0 (0.00%)	
Alcohol consumption (*n*%)			
No	36 (90.00%)	39 (97.50%)	0.356
Yes	4 (10.00%)	1 (2.50%)	

MetS, metabolic syndrome; BMI, body mass index; WC, waist circumference; HC, hip circumference; SBP, systolic blood pressure; DBP, diastolic blood pressure; SD, standard deviation; *p* value, statistical significance; *p* < 0.05, there is a statistical difference between the groups (bold values).

**Table 2:** Comparison of clinical laboratory parameters between patients with MetS and healthy controls.

Variable	Group		*t* Value	*p* Value
	MetS (Mean ± SD)	Control (Mean ± SD)		
HbA1c (%)	6.87 ± 2.2	5.29 ± 0.32	4.505	**0.001**
FG (mg/dL)	137.1 ± 67.59	87.85 ± 8.37	4.573	**0.001**
FI (uU/mL)	15.21 ± 6.57	8.84 ± 3.18	5.518	**0.001**
HOMA-IR	5.04 ± 3.04	1.92 ± 0.74	6.286	**0.001**
HDL (mg/dL)	37.81 ± 5.49	52.08 ± 11.89	−6.889	**0.001**
TC (mg/dL)	200.38 ± 35.62	169.75 ± 22.95	4.571	**0.001**
TG (mg/dL)	213.6 ± 91.46	94.08 ± 30.09	7.851	**0.001**
LDL (mg/dL)	120.52 ± 26.12	95.07 ± 18.73	5.010	**0.001**
CRP (mg/dL)	0.58 ± 0.66	0.19 ± 0.33	3.416	**0.001**
ALT (U/L)	30.15 ± 16.68	20.2 ± 9.2	3.304	**0.001**
AST (U/L)	24.6 ± 7.09	23.3 ± 7.31	0.807	0.422
Urea (mg/dL)	24.9 ± 7.11	23.11 ± 5.05	1.299	0.198
Creatinine (mg/dL)	0.76 ± 0.2	0.79 ± 0.13	−0.868	0.388
Uric acid (mg/dL)	4.93 ± 1.42	4.87 ± 1.21	0.212	0.833
Albumin (g/dL)	4.52 ± 0.3	4.43 ± 0.3	1.352	0.180
HG (g/dL)	14.51 ± 1.65	14.34 ± 1.97	0.425	0.672
Erythrocyte (10^6^/µL)	5.24 ± 0.5	5.04 ± 0.49	1.729	0.088
Leukocyte (10^3^/µL)	8.86 ± 2.35	7.48 ± 1.71	3.007	**0.004**
Lymphocyte (10^3^/µL)	2.76 ± 0.85	2.47 ± 0.79	1.556	0.124
Monocyte (10^3^/µL)	0.6 ± 0.26	0.57 ± 0.11	0.557	0.579
Neutrophil (10^3^/µL)	4.47 ± 1.24	3.86 ± 0.91	2.505	**0.014**
Platelet (10^3^/µL)	282.82 ± 50.29	267.62 ± 51.25	1.339	0.185
MCV (fL)	87.72 ± 3.82	89.91 ± 3.57	−2.657	**0.010**
TSH (mU/L)	1.66 ± 1.06	1.84 ± 1.35	−0.660	0.511
Free T3 (pg/mL)	3.29 ± 0.62	3.21 ± 0.46	0.652	0.517
Free T4 (ng/mL)	1.37 ± 0.25	1.45 ± 0.32	−1.221	0.226

MetS, metabolic syndrome; HbA1c, glycated hemoglobin; FG, fasting glucose; FI, fasting insulin; HOMA-IR, homeostatic model assessment of insulin resistance; HDL, high-density lipoprotein; TC, total cholesterol; TG, triglyceride; LDL, low-density lipoprotein; CRP, C-reactive protein; ALT, alanine aminotransferase; AST, aspartate aminotransferase; HG, hemoglobin; MCV, mean corpuscular volume; TSH, thyroid-stimulating hormone; T3, triiodothyronine; T4, thyroxine; SD, standard deviation; *p* value, statistical significance; *p* < 0.05, there is a statistical difference between the groups (Bold values).

**Table 3:** Group * BMI interaction effects on study variables.

Variable		Group		F	*p*	η^2^
		MetS	Control			
		(Mean ± SD)	(Mean ± SD)			
Group × BMI interaction effects	HIF1α	3.25 ± 2.17	1.25 ± 0.42	2.303	0.133	0.029
	BNIP3	1.23 ± 0.31	0.76 ± 0.17	0.048	0.828	0.001
	BNIP3L	0.41 ± 0.14	0.28 ± 0.09	0.011	0.917	0.000
	TG	213.6 ± 91.46	94.08 ± 30.09	3.163	0.079	0.040
	LDL	120.52 ± 26.12	95.07 ± 18.73	0.337	0.563	0.004
	HDL	37.81 ± 5.49	52.08 ± 11.89	2.544	0.115	0.032
	WC	108.1 ± 8.89	77.53 ± 13.23	0.576	0.450	0.008
	SBP	128.25 ± 10.83	114.75 ± 7.16	0.097	0.757	0.001
	DBP	82.5 ± 6.3	76.9 ± 12.85	0.397	0.531	0.005
	FG	137.1 ± 67.59	87.85 ± 8.37	0.694	0.408	0.009

MetS, metabolic syndrome; BMI, body mass index; HIF1α, hypoxia-inducible factor 1 alpha; BNIP3, Bcl-2/adenovirus E1B 19 kDa interacting protein 3; BNIP3L, BNIP3-like; TG, triglyceride; LDL, low-density lipoprotein; HDL, high-density lipoprotein; WC, waist circumference; SBP, systolic blood pressure; DBP, diastolic blood pressure; FG, fasting glucose; SD, standard deviation; F, ANCOVA analysis test value; *p* value, statistical significance; η2, partial eta squared.

## Data Availability

The datasets generated and analyzed during this study are available from the corresponding author on reasonable request.
